# Human Serum Albumin Decorated Indocyanine Green Improves Fluorescence-Guided Resection of Residual Lesions of Breast Cancer in Mice

**DOI:** 10.3389/fonc.2021.614050

**Published:** 2021-03-08

**Authors:** Zun Wang, Min Chen, Jing-Jing Liu, Rong-He Chen, Qian Yu, Gui-Mei Wang, Li-Ming Nie, Wen-He Huang, Guo-Jun Zhang

**Affiliations:** ^1^ChangJiang Scholar’s Laboratory, Medical College, Shantou University, Shantou, China; ^2^Clinical Central Research Core, Xiang’an Hospital of Xiamen University, School of Medicine, Xiamen University, Xiamen, China; ^3^Key Laboratory for Endocrine-Related Cancer Precision Medicine of Xiamen, Xiang’an Hospital of Xiamen University, Xiamen, China; ^4^Cancer Research Center, School of Medicine, Xiamen University, Xiamen, China; ^5^Cancer Center & Department of Breast and Thyroid Surgery, Xiang’an Hospital of Xiamen University, School of Medicine, Xiamen University, Xiamen, China; ^6^State Key Laboratory of Molecular Vaccinology and Molecular Diagnosis & Center for Molecular Imaging and Translational Medicine, School of Public Health, Xiamen University, Xiamen, China; ^7^Department of Pathology, Xiang’an Hospital of Xiamen University, School of Medicine, Xiamen University, Xiamen, China

**Keywords:** breast cancer, tumor margin, indocyanine green, fluorescence-guided surgery, human serum albumin

## Abstract

**Objective:**

Achieving negative resection margin is critical but challenging in breast-conserving surgery. Fluorescence-guided surgery allows the surgeon to visualize the tumor bed in real-time and to facilitate complete resection. We envisioned that intraoperative real-time fluorescence imaging with a human serum albumin decorated indocyanine green probe could enable complete surgical removal of breast cancer in a mouse model.

**Methods:**

We prepared the probe by conjugating indocyanine green (ICG) with human serum albumin (HSA). *In vitro* uptake of the HSA-ICG probe was compared between human breast cancer cell line MDA-MB-231 and normal breast epithelial cell line MCF 10A. *In vivo* probe selectivity for tumors was examined in nude mice bearing MDA-MB-231-luc xenografts and the FVB/N-Tg (MMTV-PyMT) 634Mul/J mice model with spontaneous breast cancer. A positive-margin resection mice model bearing MDA-MB-231-luc xenograft was established and the performance of the probe in assisting surgical resection of residual lesions was examined.

**Results:**

A significantly stronger fluorescence intensity was detected in MDA-MB-231 cells than MCF 10A cells incubated with HSA-ICG. *In vivo* fluorescence imaging showed that HSA-ICG had an obvious accumulation at tumor site at 24 h with tumor-to-normal tissue ratio of 8.19 ± 1.30. The same was true in the transgenic mice model. The fluorescence intensity of cancer tissues was higher than that of non-cancer tissues (58.53 ± 18.15 *vs* 32.88 ± 11.34). During the surgical scenarios, the residual tumors on the surgical bed were invisible with the naked eye, but were detected and resected with negative margin under HSA-ICG guidance in all the mice (8/8). Recurrence rate among mice that underwent resection with HSA-ICG (0/8) was significantly lower than the rates among mice with ICG (4/8), as well as the control group under white light (7/7).

**Conclusions:**

This study suggests that real-time *in vivo* visualization of breast cancer with an HSA-ICG fluorescent probe facilitates complete surgical resection of breast cancer in a mouse xenograft model.

## Introduction

Breast-conserving surgery (BCS) with adjuvant whole-breast radiation therapy is the standard care for early-stage breast cancer. Successful BCS involves removal of malignant tissues with enough surrounding normal breast tissues to ensure complete tumor excision (negative margin), while leaving enough normal tissues to preserve the breast and provide an aesthetically acceptable result. However, 20–40% patients show positive margins after BCS, leading to unwanted reoperation (1, 2). This causes patients’ anxiety and suffering, which may also compromise aesthetic results and increase infection risks and healthcare costs. In addition, margin status is a key risk factor for local recurrence and a prognostic factor for overall survival after BCS (3). A meta-analysis based on 33 studies showed that positive margins were associated with a 2-fold higher risk of local recurrence than negative margins (4).

To identify the resection margins involved and perform immediate selective margin re-excision, intraoperative margin assessment techniques have been investigated. Established methods include frozen sectioning, imprint cytology, intraoperative ultrasound, and specimen mammography (5). However, all the above techniques have their own limitations to some extent. Frozen sectioning and imprint cytology often disrupt the surgical workflow and are labor-intensive and time-consuming (6). Anatomical imaging modalities such as intraoperative ultrasound and specimen mammography could provide instant feedback of margin status but lack diagnostic accuracy (7). So far, no technique has been universally adopted for intraoperative margin assessment to the best of our knowledge. Therefore, it is imperative to develop an ideal method to identify involved margins rapidly and accurately.

Molecular fluorescence imaging coupled with contrast agent introduces a promising technique to visualize the tumor lesion, which can delineate tumor margins against normal tissues. It could provide instant feedback during surgery and augment the visual range for surgeons. For example, fluorescence-guided surgery with 5-aminolevulinic acid could make more complete resections with malignant glioma, which also improves patient outcomes (8). Near-infrared fluorescence imaging, at wavelengths in the 700–900 nm range, is particularly promising because autofluorescence from native tissue is minimal in this range, allowing a high signal-to-background ratio (9). Indocyanine green (ICG) is the only near-infrared fluorescent dye approved by the U.S. Food and Drug Administration for intravenous injections in humans. Several studies reported that intraoperative ICG fluorescence could localize non-palpable or occult breast cancer lesion and guide its excision (10, 11). Studies in mouse models of breast cancer showed that ICG is superior to visual inspection and palpation for identifying retained tumor margins (12, 13). But in the clinical application for fluorescence-guided surgery, ICG seemed to be less reliable for identifying positive margins. The potential explanation is that ICG cannot bind to specific ligands within the target tumor but spread to peritumoral tissues (13).

It is desirable to engineer ICG that binds selectively to tumors by combining it with human serum albumin (HSA). HSA plays an active role in tumor nutrition and demonstrates an obvious increased uptake by solid tumors (14). The tumor targeting makes HSA a promising carrier for cancer bioimaging and drug delivery (15, 16). It has been reported that HSA-ICG nanoparticles can be successfully applied to photothermal and photodynamic therapy (17), and tumor diagnosis based on fluorescence imaging and photoacoustic imaging (18, 19) in the literature. In the present study, we focused on its performance in guiding surgical removal of breast cancer in a mouse model. ICG was conjugated with HSA as a contrast agent for fluorescence imaging of breast cancer. Then, we evaluated the tumor selectivity of the HSA-ICG probe *in vitro* and *in vivo*. We subsequently used a mice model of positive margin resection with MDA-MB-231-luc xenograft to investigate whether HSA-ICG fluorescence guidance could detect tumor deposits. Finally, we compared the local recurrence rate and overall survival rate after surgery with HSA-ICG fluorescence guidance or not.

## Materials and Methods

### Preparation and Characterization of Human Serum Albumin-Indocyanine Green

ICG-NHS (Xinyan Bomei Bio, Xi’an, China) was conjugated to HSA (Solarbio, Beijing, China) using a previously published protocol with minor modifications (20). Briefly, HSA was incubated with ICG at a molar ratio of 1:5 in 0.1 M Na2HPO4 (pH 8.5) at room temperature for 2 h. The mixture was purified on a Sephadex G50 column (PD-10; GE Healthcare, Piscataway, USA). The morphology of the resulting HSA-ICG conjugate was observed by G2 F30 Twin transmission electron microscopy (FEI/TECNAI, Hillsboro, USA). The molecular weight of the conjugate was determined using SDS-PAGE and Coomassie Brilliant Blue staining. Gels were scanned at 800 nm using the Odyssey CLx scanner (LI-COR Bio, Lincoln, USA) to determine the intactness of HSA-ICG. The absorption spectrum of the conjugate was measured using a spectrophotometer (Thermo Multiskan GO, Thermo Fisher Scientific, Waltham, USA). The fluorescence emission spectrum was obtained after excitation at 745 nm using a fluorimeter (TECAN SPARK, Mannedorf, Switzerland). Protein concentration of the samples was determined using bicinchoninic acid assay (Solarbio, Beijing, China). The concentration of ICG of the probe was also measured by absorption with the above spectrophotometer to calculate the number of fluorophores conjugated to HSA. The purified HSA-ICG probes were stored in 0.1M phosphate-buffered saline (PBS) at 4°C.

### Cell Cultures

Human breast cancer cell line MDA-MB-231 and non-cancerous breast epithelial cell line MCF 10A were purchased from the American Type Culture Collection (Rockville, USA). The MDA-MB-231-luc cell line was purchased from ZQXZ Biotechnology (Shanghai, China). These cell lines were cultured according to the vendors’ recommendations.

### Uptake and Cytotoxicity of Human Serum Albumin-Indocyanine Green *In Vitro*

MDA-MB-231 cells were seeded in six-well plates at a density of 2 × 10^5^ cells per well, and incubated overnight for cell attachment. Then the cells were treated with HSA-ICG (at a final ICG concentration of 10 µg/ml) alone or in the presence of excess HSA (10 mg/ml) for 8 h. Cells were collected at 1, 2, 4, and 8 h, washed with cold PBS (1–4°C). Then the signal intensity of HSA-ICG was measured by flow cytometry (BD, Franklin Lakes, USA).

To investigate whether HSA-ICG is selectively taken up by cancer cells, MDA-MB-231 cells or MCF 10A cells were seeded at a density of 1 × 10^5^ cells per well and cultured for 24 h. Afterward, the cells were incubated in fresh medium containing HSA-ICG (10 µg/ml) at 37°C for 4 h. Cells were washed with cold PBS and then fixed in 4% paraformaldehyde and nuclei were stained with DAPI. The internalization of HSA-ICG was observed using a confocal laser scanning microscope (FV1000, Olympus, Tokyo, Japan).

Cytotoxicity of HSA-ICG against MDA-MB-231 cells was measured by incubating them for 24 h in the presence of the conjugate at concentrations of 1.25–40 μg/ml. The viability was measured using a standard CCK8 assay (Promega, Beijing, China).

### Real-Time PCR Analysis of Secreted Protein, Acidic And Rich In Cysteine Expression

Total RNA of MDA-MB-231 cells and MCF 10A cells was extracted and reverse-transcribed using Takara kits (Takara, Beijing, China) according to the manufacturer’s instructions. Real-time PCR was performed with SYBR Green qPCR Master Mix (Thermo Fisher Scientific, Waltham, USA) using a CFX96 Real-time PCR Detection System machine (Bio-Rad, Hercules, USA). SPARC primers were synthesized by Brogene Biotechnology (Xiamen, China). The primers for the amplification of SPARC were as follows: forward, 5’-TGAGGTATCTGTGGGAGCTAATC-3’; and reverse, 5’-CCTTGCCGTGTTTGCAGTG-3’. We normalized Ct values to those of beta-actin and calculated relative expression using the 2_−ΔΔCt_ method.

### Xenograft Models and Fluorescence Imaging

Animal experiments were approved by the Institutional Animal Care and Use Committee of Xiamen University, which were conducted accordance to relevant guidelines. Female BALB/c nude mice (6 weeks old, Charles River Labs, Beijing, China) received subcutaneous injection of MDA-MB-231-luc cells (5 × 10^6^) in the right hind limb. Tumor volume was calculated as [π/6 × length × (width)^2^].

When tumor volume reached 80 mm^3^, HSA-ICG at doses equivalent to 1 mg ICG per kg or free ICG at 1 mg/kg was injected through the tail vein (n = 3 for each group). Mice were anesthetized with 2% isoflurane and fluorescence was imaged *in vivo* using an IVIS Lumina II imaging system (Perkin Elmer, Waltham, USA) at different time points (0, 3, 6, 9, 12, 24, 36, 48, 72, 96, and 120 h) after injection. Blood samples were also collected to determine fluorescence signal using a fluorimeter (TECAN SPARK). *Ex vivo* imaging was conducted at 24 h to determine fluorescence in the heart, liver, spleen, lungs, kidneys, and tumor. Tumor-to-background ratio (TBR) was defined as the fluorescence intensity in the tumor divided by the intensity in the upper limb.

### Fluorescence Imaging Using Human Serum Albumin-Indocyanine Green in a Mouse Model of Spontaneous Breast Cancer

FVB/N-Tg(MMTV-PyMT)634Mul/J mice (Jackson Laboratory) were used to model orthotopic breast cancer. These mice spontaneously developed invasive breast carcinoma at a mean age of 53 days (21). At 7–9 weeks old, 10 mice were injected with HSA-ICG (1 mg/kg) through the tail vein. Twenty-four hours later, the combined 4^th^ and 5^th^ mammary glands (n = 20) were divided into four quadrants, each quadrant of the gland was removed sequentially. Fluorescence images of the mice were obtained before and after removal of each quadrant of the gland. All resected tissues were also analyzed by fluorescence imaging and histology. The latter was performed by an experienced pathologist according to mammary pathology of genetically engineered mice as described (22). The gland tissues were diagnosed as malignant or not based on histology. At last we correlated the pathology results with the fluorescence images of the resected tissues.

### Establishment of a Positive-Margin Resection Model

Mice bearing MDA-MB-231-luc tumors of 600–700 mm^3^ underwent resection under 2% isoflurane anesthesia. To establish a positive-margin resection model, 95% of the tumor mass was resected using a blunt dissection technique under white light (23). Then all the surgical bed was examined independently by two investigators. The mice were excluded if residual tumor was visible to the naked eye. At the same time, all the mice were examined to confirm the presence of residual tumor by bioluminescence imaging with 150 mg/kg D-luciferin (Perkin Elmer, Waltham, USA) *via* intraperitoneal administration. All the remaining animals thought to be disease-free were then scanned using the hand-held near-infrared imaging system.

### Residual Breast Cancer Tissue Resection Under the Human Serum Albumin-Indocyanine Green Fluorescence Guidance

At 24 h prior to surgery, the mice were received an intravenous injection of HSA-ICG (1 mg/kg of ICG, n = 10). Fluorescence imaging of tumor was performed prior to surgery using a hand-held near-infrared imaging system (Mingde Biotech, Langfang, China) with filters of 760/30 nm for excitation and 820/20 nm for emission. When the positive-margin resection model was established, two mice were excluded because of the visible residual tumor and eight remaining mice were used for the next surgery. Tissues with high fluorescence signals in the tumor bed were removed. Then, standard pathological procedures were used to determine whether residual foci exist in the resected fluorescence tissues with free margin. In addition, a margin of remaining nonfluorescent tissue was also removed and processed as control. Margins were defined as positive if tumor cells extended to the outside surface of the resected tissue.

### Fluorescence Imaging of Specimen

After excision of the specimen, *ex vivo* imaging was performed with the hand-held near-infrared imaging system. Then, all the specimen was fixed in formalin and embedded in paraffin blocks and made into10-µm-thick tissue sections. We scanned the slides using the Odyssey CLx scanner (LI-COR Bio, Lincoln, USA). Microscopic assessment of the slides derived from HSA-ICG fluorescence signal was made to demonstrate the tracer distribution at a cellular level.

### Fluorescence Image Analysis Related to Fluorescence-Guided Surgery

Intraoperative fluorescence images were recorded at three predefined time points: before the operation, before and after removal of tissue with fluorescence signals. Fluorescence images of specimens and slides were also recorded. Heatmap were created based on gray-scaled fluorescence images using MATLAB (MathWorks, Natick, USA). Fluorescence images as well as the regions of interest representing different tissue components were imported into ImageJ (National Institutes of Health, USA). Mean fluorescence intensity (MFI) was measured in arbitrary units for tumor and normal tissue *in vivo*, *ex vivo* and at tissue slice. TBR was defined as the MFI of tumor tissues divided by the MFI of surrounding normal tissues.

In addition, tumor lysates from mice that underwent fluorescence-guided surgery were analyzed by SDS-PAGE to confirm the presence of HSA-ICG in the tumor. The gel was scanned using the Odyssey CLx scanner at 800 nm.

### Local Recurrence and Survival After the Fluorescence-Guided Surgery

During the fluorescence-guided surgery, HSA-ICG or free ICG (1 mg/kg) or saline as control was injected 24 h before the operation (n = 10 per group). After the establishment of positive-margin resection model, all the residual tumor mice (eight in the HSA-ICG group, eight in the ICG group, seven in control group) undergone fluorescence-guided surgery. After the surgery, mice were returned to their home cages. Local recurrence was observed every 2 days for 2 weeks and survival for 5 weeks. Mice were euthanized if weight loss exceeded 20% or recurrence tumor volume reached 1,500 mm^3^.

### Evaluation on Biosafety of Human Serum Albumin-Indocyanine Green

Healthy female FVB/NCrl mice (4–6 weeks old, Xiamen University) were randomly divided into two groups (n = 15 for each group). Mice in the HSA-ICG group received the injection of HSA-ICG probe (6 mg ICG per kg), while mice in the control group received the injection of saline solution. The mice were sacrificed on days 1, 7, and 30 (n = 5 per time point) to collect blood samples and major organs (heart, liver, spleen, lung, and kidney). The blood samples were measured for serum biochemical markers, including aspartate aminotransferase, alanine aminotransferase, albumin, total protein, total bilirubin, creatinine, and blood urea nitrogen. The major organs were stained with hematoxylin and eosin for histological analysis. During the observation period, the weight of the mice (n = 5) in each group was recorded every other day.

### Statistical Analysis

We used GraphPad Prism 7.0b (GraphPad Software Inc., San Diego, USA) for statistical analyses. Quantitative results were presented as mean ± standard deviation. Differences among more than two groups were assessed for significance using one-way ANOVA followed by the Duncan multiple comparisons test. Differences between two groups were assessed using the Student’s *t* test. Inter-group differences in postoperative recurrence and overall survival were assessed using the log-rank test. A two-sided P value of less than 0.05 was considered to be statistically significant.

## Results

### Human Serum Albumin-Indocyanine Green Characterization

Negative staining of transmission electron microscopy images revealed that the diameters of HSA and HSA-ICG were 5.38 ± 0.92 and 5.69 ± 0.82 nm, respectively (**Figure S1A**). Thus, the conjugation of ICG to HSA almost had no effect on the overall size. The apparent molecular weight of HSA-ICG was only slightly higher than that of HSA, and SDS-PAGE did not detect any substantial aggregates of HSA (**Figure S1B**). Furthermore, fluorescence scanning showed that the fluorescence signal coincided with the location of HSA-ICG on the gel, while free ICG was located at the bottom of the gel (**Figure S1B**). This indicated that the HSA-ICG conjugate was stable and did not dissociate during SDS-PAGE. ICG alone and HSA-ICG showed similar absorption peaks (788 *vs* 795 nm) and fluorescence emission peaks (820–825 nm) (**Figures S1C, D**). The slight red shift in absorption (~7 nm) further suggest the successful conjugation of ICG to HSA. The purified HSA-ICG conjugate was estimated to contain 3.5 ICG per 1 HSA.

### Uptake of Human Serum Albumin-Indocyanine Green *In Vitro*

**Figure 1:**
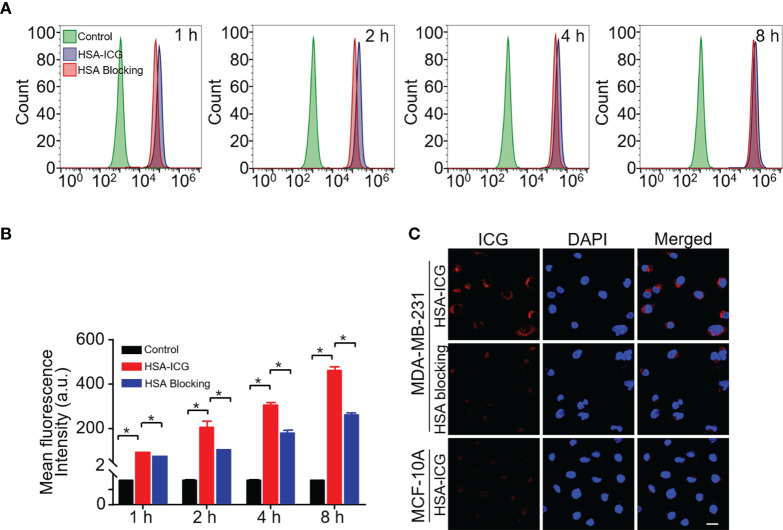
Cell uptake of HSA-ICG. **(A)** Histogram showing flow cytometry based on fluorescence in MDA-MB-231 cells incubated for 1, 2, 4, or 8 h with HSA-ICG (10 µg/ml) alone or in the presence of excess HSA (10 mg/ml). **(B)** Mean fluorescence intensity from the flow cytometry in panel A at selected time points (n = 3, one-way ANOVA, *P < 0.05). **(C)** Confocal laser scanning micrographs of MDA-MB-231 and MCF 10A after 4 h incubation with HSA-ICG. Scale bar = 20 µm.

Fluorescence signals in MDA-MB-231 cells detected increased when incubated with HSA-ICG probes for 1, 2, 4, or 8 h, while the signals decreased when excess HSA was added (**Figure 1A**). This suggested that MDA-MB-231 cells internalized HSA-ICG in a time-dependent manner, the process of which was weakened in the presence of excess HSA. Quantitatively, the mean fluorescence intensity was significantly higher in HSA-ICG treated cells than in HSA-ICG treated cells with HSA blocking at selected time points (**Figure 1B**, one-way ANOVA, *P* < 0.05). Moreover, the probe mainly localized in the cytoplasm of MDA-MB-231 cells. On the contrary, normal breast epithelial MCF 10A cells internalized little HSA-ICG (**Figure 1C**). Relative mRNA expression of SPARC was significantly higher in breast cancer MDA-MB-231 cells than in human breast epithelial cells (**Figure S2**, unpaired Student’s *t* test, *P* < 0.05).

### *In Vivo* and *Ex Vivo* Fluorescence Imaging

**Figure 2:**
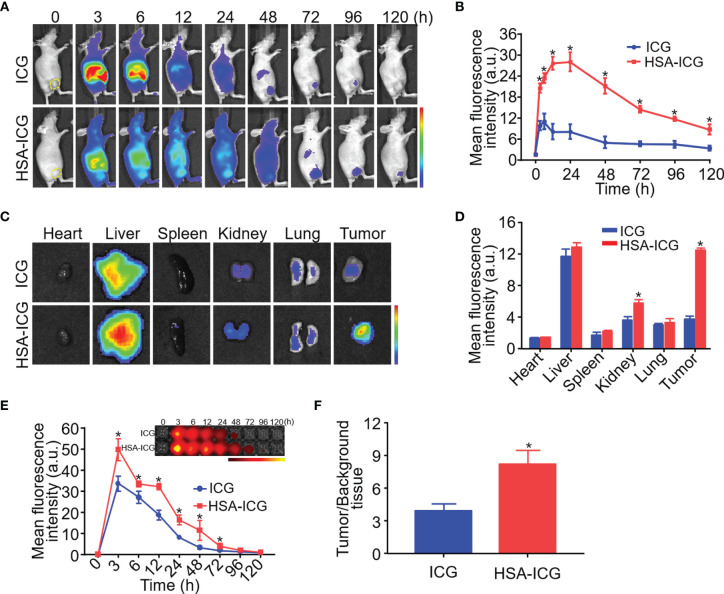
*In vivo* and *ex vivo* fluorescence imaging of HSA-ICG in MDA-MB-231 tumor-bearing mice. **(A)** *In vivo* fluorescence images at indicated time points after intravenous injection of ICG or HSA-ICG (1 mg ICG per kg). Tumor tissue is delineated with a dashed line in the “0” images at far left. **(B)** Semi-quantitative analysis of *in vivo* fluorescence intensity of tumors. **(C)** Fluorescence images from organs and tumors excised 24 h after the injection of ICG or HSA-ICG. **(D)** Semiquantitative analysis of fluorescence signals from the samples in panel **(C)**. **(E)** Fluorescence images (inset) and semiquantitative analysis of fluorescence in blood sampled at different times after intravenous injection of the probe. **(F)** Tumor-to-background tissue ratios obtained at 24 h after injection of HSA-ICG or ICG. Background tissue was from the upper limb. Each group n = 3, (unpaired Student’s *t* test, *P < 0.05).

When mice received ICG alone, the fluorescence signal appeared primarily in liver 3 h post-injection, but it was detected mainly in liver and tumor at 48 h, albeit considerably weaker. In contrast, the HSA-ICG fluorescence signal retained in the body for a longer time, as shown by a gradual increase that finally peaked at the tumor site at about 24 h (**Figures 2A, B**). *Ex vivo* fluorescence images of dissected organs and tumors 24 h post-injection confirmed that most free ICG accumulated in the liver, whereas enhanced fluorescent signals could be detected in liver, kidney, and tumor of HSA-ICG treated mice (**Figures 2C, D**). Compared with ICG-treated mice, blood from HSA-ICG treated mice showed significantly higher fluorescence intensity within 72 h after injection (**Figure 2E**). Furthermore, TBR reached the peak at 24 h in HSA-ICG treated mice, significantly higher than in ICG-treated mice (8.19 ± 1.30 *vs* 3.87 ± 0.68, unpaired Student’s *t* test, *P* < 0.05) (**Figure 2F**, **Figure S3**).

### Human Serum Albumin-Indocyanine Green Imaging in a Mouse Model of Spontaneous Breast Cancer

**Figure 3:**
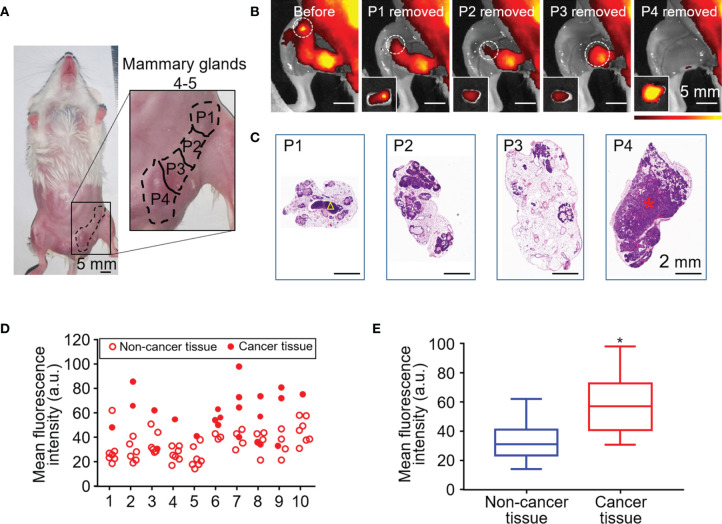
HSA-ICG fluorescence imaging in a mouse model of spontaneous breast cancer. **(A)** Picture of mammary glands 4 and 5 divided into four approximately equal quadrants (P1–4), which were resected sequentially 24 h after the injection of HSA-ICG (1 mg/ml). **(B)** Representative fluorescence images before resection (leftmost) and after each sequential resection. Tissues resected (P1–4) are marked in white circles, and the corresponding *ex vivo* fluorescence images of excised tissues are shown as *insets* on the lower left. **(C)** Histological examination of resected pieces (P1–4), corresponding to the fluorescence images in panel **(B)** A yellow triangle in P1 marks a lymph node; a red asterisk in P4, a cancer lesion. **(D)** The fluorescence intensity distribution of the resected mammary gland tissues (P1–P4) in every case (n = 10). **(E)** Mean fluorescence intensity of 23 resected cancer tissues and 57 non-cancer tissues pooled from the 10 mice in panel **(D)** (unpaired Student’s *t* test, *P < 0.05).

Fluorescence imaging was performed before and after surgical resection of breast tissue in a mouse model of spontaneous breast cancer (**Figures 3A, B**). Resected samples (n = 80) were also examined histologically (**Figure 3C**) and classified as non-cancerous (n = 57) or cancerous (n = 23). The fluorescence intensity of all the samples were assessed in each mouse (**Figure 3D**). MFI of cancerous tissue was significantly higher than that of non-cancerous tissue (58.53 ± 18.15 *vs* 32.88 ± 11.34, unpaired Student’s *t* test, *P* < 0.05) (**Figure 3E**).

### Residual Breast Cancer Resection Guided by Human Serum Albumin-Indocyanine Green Fluorescence

**Figure 4:**
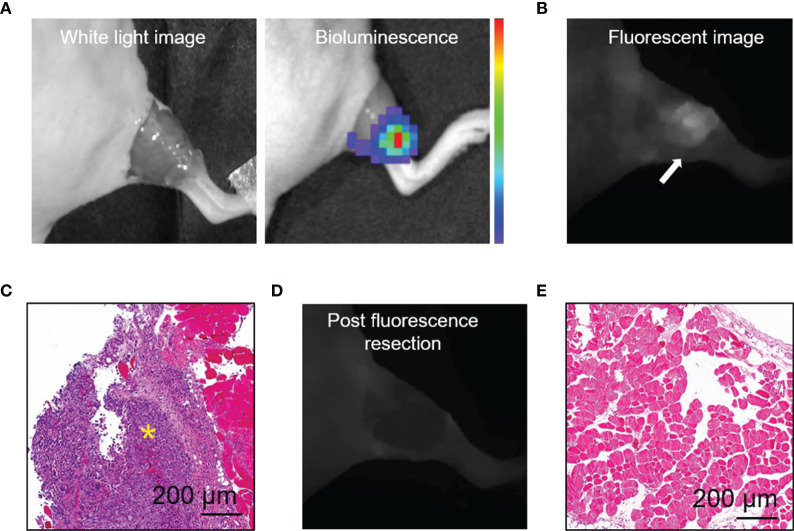
HSA-ICG enabled fluorescence-guided resection of residual lesions in MDA-MB-231-luc tumor-bearing mice. **(A)** Establishment of the model of positive-margin resection. The residual cancer was not clearly seen by the unaided eye (left), and was confirmed by bioluminescence imaging (right). **(B)** Fluorescence image of the tumor bed showed residual cancer undetectable by white light. **(C)** Hematoxylin and eosin staining of the resected tissue from **(B)**. A yellow asterisk marks the tumor. **(D)** Fluorescent image of the tumor bed following fluorescence-guided resection, indicating the absence of fluorescence signal. **(E)** Tissues on the tumor bed from **(D)** were collected and examined histologically to confirm the absence of residual tumor.

We established a residual tumor model using MDA-MB-231-luc tumor-bearing mice whose xenografts were missed during surgery under white light alone. We confirmed the presence of tumor deposits in the tumor bed by bioluminescence (**Figure 4A**). Next, following the guidance of real-time fluorescence imaging, we observed the aggregated fluorescent signal in the tumor bed. Subsequently, the tissues with high fluorescence signal (**Figure 4B**) were resected and postoperative pathology examination validated the presence of cancer foci (**Figure 4C**). The fluorescence images obtained after the excision process indicated the removal of tissues with high fluorescence signal on the surgical bed (**Figure 4D**). Histology of tissues from the area confirmed the absence of residual tumors (**Figure 4E**).

### Quantitative Analysis of Human Serum Albumin-Indocyanine Green Fluorescence Imaging During Surgery

**Figure 5:**
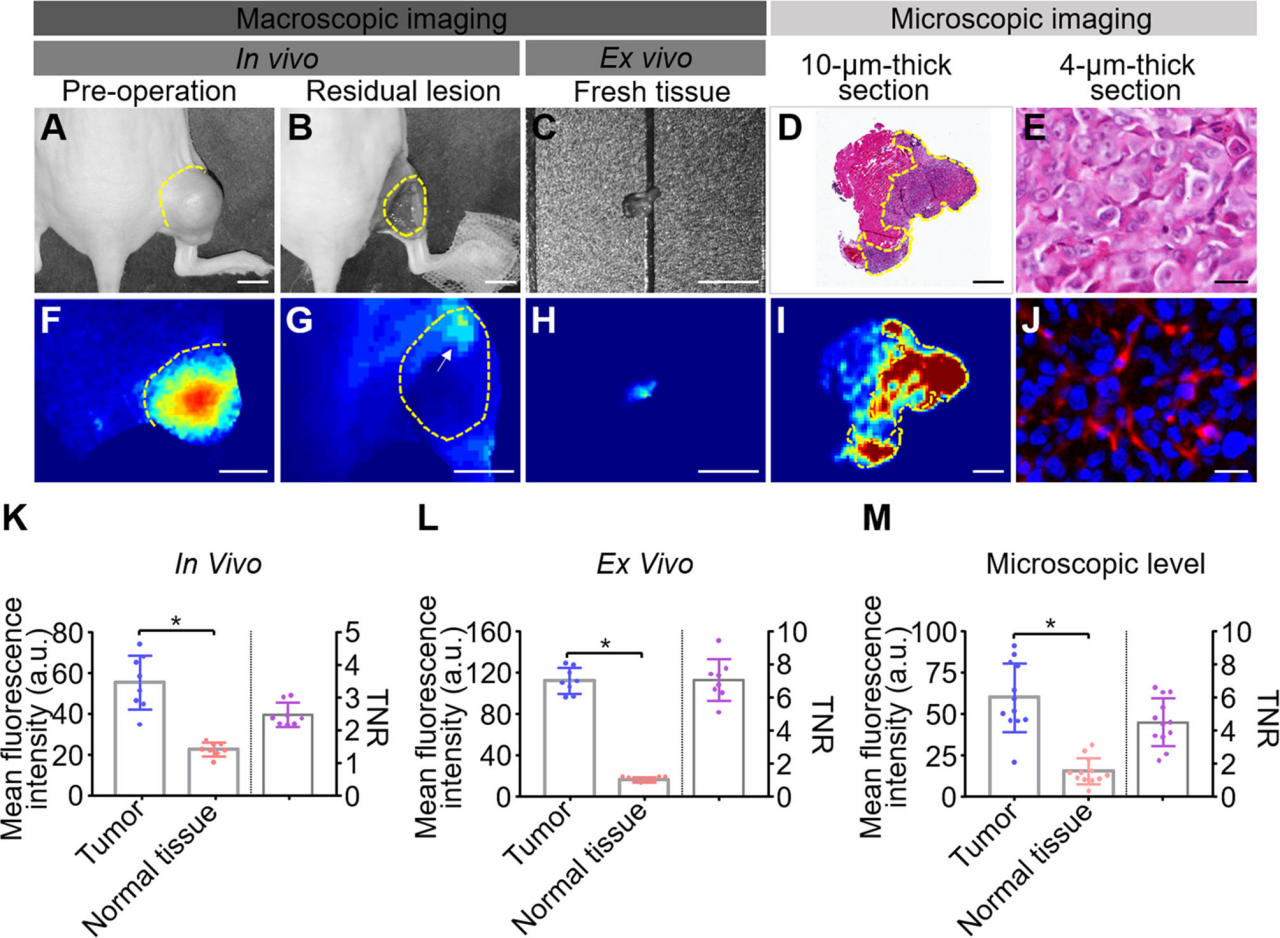
Quantitative analysis of fluorescence images from fluorescence-guided surgery using HSA-ICG. **(A–J)** Correlation of fluorescence signals with pathology during fluorescence-guided surgery. **(A, F)** white-light images and corresponding fluorescence images of MDA-MB-231 tumors prior to surgery. **(B, G)** the corresponding images of the surgical bed of residual cancer model under HSA-ICG guidance. The yellow arrow in **(G)** points to the local aggregated fluorescence signal on the surgical bed. **(C, H)** *ex vivo* images of tissue resected from **(B)**. **(D)** hematoxylin and eosin staining of resected tissue, while **(I)** the corresponding fluorescence image. **(E, J)** the tracer at much higher cellular resolution. **(K–M)** Quantitative analysis of fluorescence signals of tumor and surrounding normal tissues *in vivo* **(K)**, *ex vivo* **(L)**, and in pathology slices **(M)**. Mean fluorescence intensity of normal tissues (red) and tumor tissues (blue) is depicted on the left y-axis, while the right y-axis shows the mean fluorescence intensity ratio of tumors to normal tissues. Tumors are outlined with a dashed line. Scale bars in all panels = 5 mm except **(D, I)**, where the scale bar = 500 µm; and **(E, J)**, where the scale bar = 25 µm. (paired Student’s *t* test, * *P* < 0.05).

Intraoperative and postoperative fluorescence images were analyzed to determine the ability of HSA-ICG in fluorescence-guided surgery for residual disease resection (**Figures 5A–J**) as recommended (24). The MFI were significantly higher for tumor tissues than surrounding normal tissues both *in vivo* (54.93 ± 13.37 *vs* 22.25 ± 3.32, paired Student’s *t* test, *P* < 0.05, TBR 2.45 ± 0.36) and *ex vivo* (111.68 ± 12.59 *vs* 16.20 ± 2.54, paired Student’s *t* test, *P* < 0.05, TBR 7.03 ± 1.27) (**Figures 5K, L**). This demonstrates that fluorescence imaging *ex vivo* can also be a useful diagnostic test to confirm successful removal. Histology of tissue sections confirmed significantly higher MFI in tumors than normal tissues (60.03 ± 20.88 *vs* 15.13 ± 7.94, paired Student’s *t* test, *P* < 0.05, TBR 4.50 ± 1.45) (**Figure 5M**). Analysis of tumor lysates confirmed that the HSA-ICG conjugate was intact in tumor tissue (**Figure S4**).

### Tumor-Free Surgical Margins Following Resection With Human Serum Albumin-Indocyanine Green Guidance

**Figure 6:**
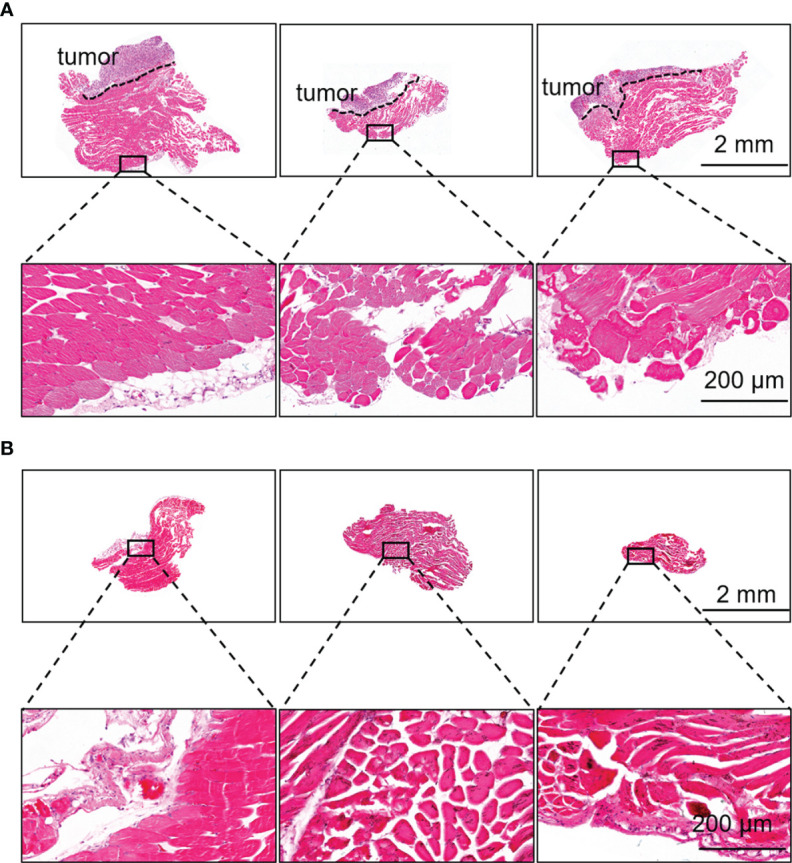
Fluorescence-guided surgery based on HSA-ICG enabled complete removal of residual breast cancer lesions. **(A)** Representative hematoxylin and eosin staining of resected tissues that showed fluorescence signal during surgery and a negative margin. Dashed lines demarcate the tumor. Rectangles indicate the location of the higher-magnification views of the tissue margins, shown in the lower row. **(B)** Representative hematoxylin and eosin staining of the tissues on the surgical bed after surgery to confirm the absence of residual tumor.

During the guidance of HSA-ICG fluorescent probes, there were multiple sites of fluorescence adjacent to a single tumor bed. A total of 12 such sites in eight animals were detected and resected, and confirmed by histology to be cancerous. Diameters of tumor foci on the slices of resected tissues (n = 12) ranged from 1.16 to 3.37 mm (mean, 2.19 mm). Histology showed that the margins of tissues resected under fluorescence guidance were tumor-free (**Figure 6A**), and that no residual tumor was detectable in the resected surgical bed in any of the eight animals (**Figure 6B**).

### Decreased Local Recurrence and Improved Overall Survival With Human Serum Albumin-Indocyanine Green Guided Surgery

**Figure 7:**
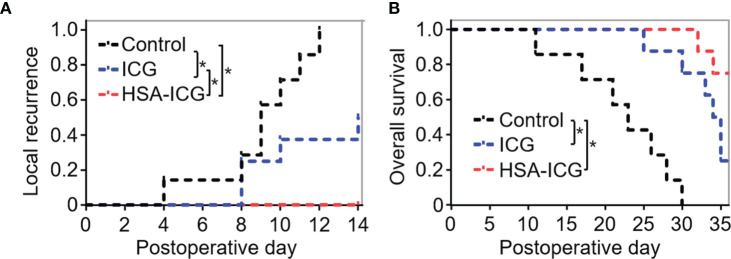
Local recurrence and overall survival of MDA-MB-231-luc tumor-bearing mice after fluorescence-guided surgery. **(A)** Local recurrence within 2 weeks for mice that underwent resection under the guidance with HSA-ICG (n = 8 animals), ICG (8) or the control group (7). **(B)** Overall survival of the same mice as **(A)**. (log-rank test, *P < 0.05).

Next, local recurrence and overall survival was observed to assess the effectiveness of HSA-ICG fluorescence imaging. Until 2 weeks after surgery, none of the eight HSA-ICG-treated animals showed local recurrence, compared to 50% of the ICG-treated mice (4/8) and all seven control mice (**Figure 7A**). Overall survival differed in the same way across the three groups: 75% of the HSA-ICG-treated animals (6/8) survived within 5 weeks after operation, compared to only 25% of the ICG-treated animals (2/8) (**Figure 7B**). All control mice died from residual disease within 30 days after surgery. The HSA-ICG group showed significantly better overall survival than the control group (log-rank test, * *P* < 0.05) and a trend of better survival compared with the ICG group.

### Human Serum Albumin-Indocyanine Green Biosafety

The CCK8 assays revealed that HSA-ICG did not significantly decrease MDA-MB-231 cell viability for 24 h, even at concentrations up to 40 μg/ml (**Figure S5**). In the HSA-ICG-treated mice, there was neither obvious damage nor inflammation in the heart, liver, spleen, lung, and kidney on the 1st, 7th, and 30th days (**Figure S6A**). HSA-ICG also did not seem to substantially alter serum biomarkers of key organ function (**Figure S6B**). HSA-ICG-treated group and the control group showed similar trends in body weight during the 30 days after tracer injection (**Figure S6C**).

## Discussion

It is essential to determine the margin status accurately during breast-conserving surgery to avoid reoperation and reduce the risk of local recurrence. Fluorescence-guided surgery images the tumor and detects residual tumor in real-time, thus providing critical guidance to the surgeon. Here we show that conjugating ICG to HSA allows to selectively accumulate in MDA-MB-231-luc xenograft tumors, where it can guide tumor removal to ensure negative margins, leading to reduced recurrence and improved overall survival.

By conjugating HSA with ICG, we developed and validated an optical imaging probe (HSA-ICG) that could accumulate selectively in MDA-MB-231-luc xenografts and the FVB/N-Tg spontaneous breast cancer lesion of (MMTV-PyMT) 634Mul/J mouse model. The molecular weight of HSA-ICG is approximately 67 kDa after the conjunction, which subsequently results in an accumulation of macromolecules (>40 kDa) within the tumor interstitium known as the enhanced permeation and retention effect (14, 25). Moreover, it also has been reported that tumors uptake albumin as a source of energy actively for their accelerated growth, by breaking down albumin into its component amino acids in lysosomes (26). In addition, receptor-mediated albumin uptake pathways by albumin binding proteins were also involved, such as membrane-associated 60 kDa glycoprotein and “secreted protein, acidic and rich in cysteine” (SPARC) (27). In previous studies, the expression level of SPARC was found to be higher in human breast cancer tissue when compared with healthy breast tissue (28–30). In the present study, we also detected that SPARC was expressed at higher levels in breast cancer MDA-MB-231 cells than in normal breast epithelial MCF 10A cells. All the reasons above may elucidate the mechanism of selective accumulation of HSA-ICG in tumors.

In several previous studies, ICG with HSA premixing by the absorption of ICG to HSA was used for near-infrared fluorescence imaging of sentinel lymph nodes (SLN), but no direct benefit was found with this probe for SLN mapping in preclinical and clinical trials (31, 32). It may be dissociated ICG from the complex due to low affinity of albumin compared with other serum protein, α1-lipoprotein, and γ-globulin (33). In the present study, HSA was conjugated with ICG derivatives (ICG-NHS ester), which could rapidly and high-specifically react to primary amine (-NH2) on HSA without altering the cyanine structure important for NIR absorption. Similar method was used with ICG derivatives (ICG-Sulfo-OSu) in previous study (19). Moreover, we confirmed that HSA-ICG was intact and present in tumor tissue by comparing the height of the band of the tumor lysates with the lane containing diluted HSA.

In the present study, we demonstrated that HSA-ICG fluorescence imaging was superior to ICG and naked eye for the detection of positive tumor margins in a surgical mice model for residual tumor. A mice model of MDA-MB-231-luc xenograft retained disease (12, 13, 23), in which the tumor deposits were not visible but could be detected by bioluminescence, was chosen to provide a scenario for examining the hypothesis that HSA-ICG can aid in identifying and completely resecting small foci of residual cancer. When using HSA-ICG to guide surgical resection, the signal on the surgical bed was sufficiently strong to be seen easily and to guide surgery in real-time with TNR approximately 2.5. During specimen imaging procedure, fluorescence imaging of the fresh surgical specimen and the pathology slides showed TNR with 7.03 and 4.50, which can provide the pathologist to outlines tumor tissue quickly and precisely. Most importantly, we confirmed the correlation of the fluorescence signal of the resected tissue with final histopathology. Furthermore, compared with surgery guided by ICG or visual inspection, HSA-ICG fluorescence-guided surgery resulted in significantly lower recurrence rate within14 days. The recurrence rate with ICG in our study (four of eight mice) was higher than the rate reported in a previous study (two of 22 mice) (12). It may be related with the usage dose of ICG, which was higher in previous study (7 *vs* 1 mg ICG per kg).

Our mice model suggests that fluorescence-guided surgery based on HSA-ICG provides a promising solution of negative margin and reduces local recurrence rates. however, small-animal models of tumor surgery could not fully reflect the complexities of human breast cancer therapy, it remains to be validated in patients. Interestingly, we found the lymph nodes surrounding the xenografts were highlighted during fluorescence-guided surgery (data not shown). Therefore, future work should also examine whether our approach can help map lymphatic metastasis, as suggested for HSA-ICG nanoprobes (18). However, specific labeling of such metastases must be established, since HSA in the tumor microenvironment is cleared *via* the lymphatic system through a natural recycling mechanism.

One limitation of our approach is that the relatively shallow penetration depth in fluorescence imaging may limit more complex resections, in which the target lesion lies behind other tissues or satellite lesions. We found that HSA-ICG can facilitate photoacoustic detection of tumors (data not shown), and this modality can penetrate down to several centimeters (34). We are exploring the use of photoacoustic imaging and HSA-ICG to detect residual lesions.

Despite these limitations, our study shows that fluorescence imaging by HSA-ICG can provide real-time imaging of tumors with high TBR, enable the surgeon to judge the completeness of resection of tumor lesion. With high biocompatibility and minimal toxicity, HSA-ICG fluorescence guidance is promising for further clinical translation in primary breast cancer patients.

## Data Availability Statement

The original contributions presented in the study are included in the article/**Supplementary Material**. Further inquiries can be directed to the corresponding authors.

## Ethics Statement

The animal study was reviewed and approved by the Institutional Animal Care and Use Committee of Xiamen University.

## Author Contributions

G-JZ designed and directed the study and finalized the manuscript. L-MN directed the study. ZW coordinated and performed the study and drafted the manuscript. MC coordinated the study. J-JL cultured the cells, raised the mice, and conducted the experiments. R-HC conducted the statistical analysis for the data. QY performed the image processing. W-HH participated in the section of the surgery navigation. G-MW performed the pathological evaluation. All authors contributed to the article and approved the submitted version.

## Funding

The present study was supported by the Natural Science Foundation of China (Grant No. 91859120), the Natural Science Foundation of Guangdong Province (Grant No. 2016A030312008), the Natural Science Foundation of Fujian Province of China (Grant No. 2020J01015), Fujian Major Scientific and Technological Special Project for Social Development (Grant No. 2020YZ016002), the Start-Up Fund for High-Talents from Xiamen University (G-JZ), and the Start-Up Fund for High-Talents from Xiang’an Hospital of Xiamen University (Grant No. PM201809170013), and the Science and Technology Plan Project of Shenzhen (No. JCYJ20160429172031572).

## Conflict of Interest

The authors declare that the research was conducted in the absence of any commercial or financial relationships that could be construed as a potential conflict of interest.

The reviewer NW declared a shared affiliation with one of the authors, ZW to the handling editor at time of review.
