# Supplementary material for: Human Serum Albumin Decorated Indocyanine Green Improves Fluorescence-Guided Resection of Residual Lesions of Breast Cancer in Mice
Source: Front Oncol. 2021 Mar 8;11:614050. doi: 10.3389/fonc.2021.614050 (PMC7983674; doi:10.3389/fonc.2021.614050)
Supplement: Supplementary file 1 [file DataSheet_1.docx]

Supplementary Material

## Supplementary Figures


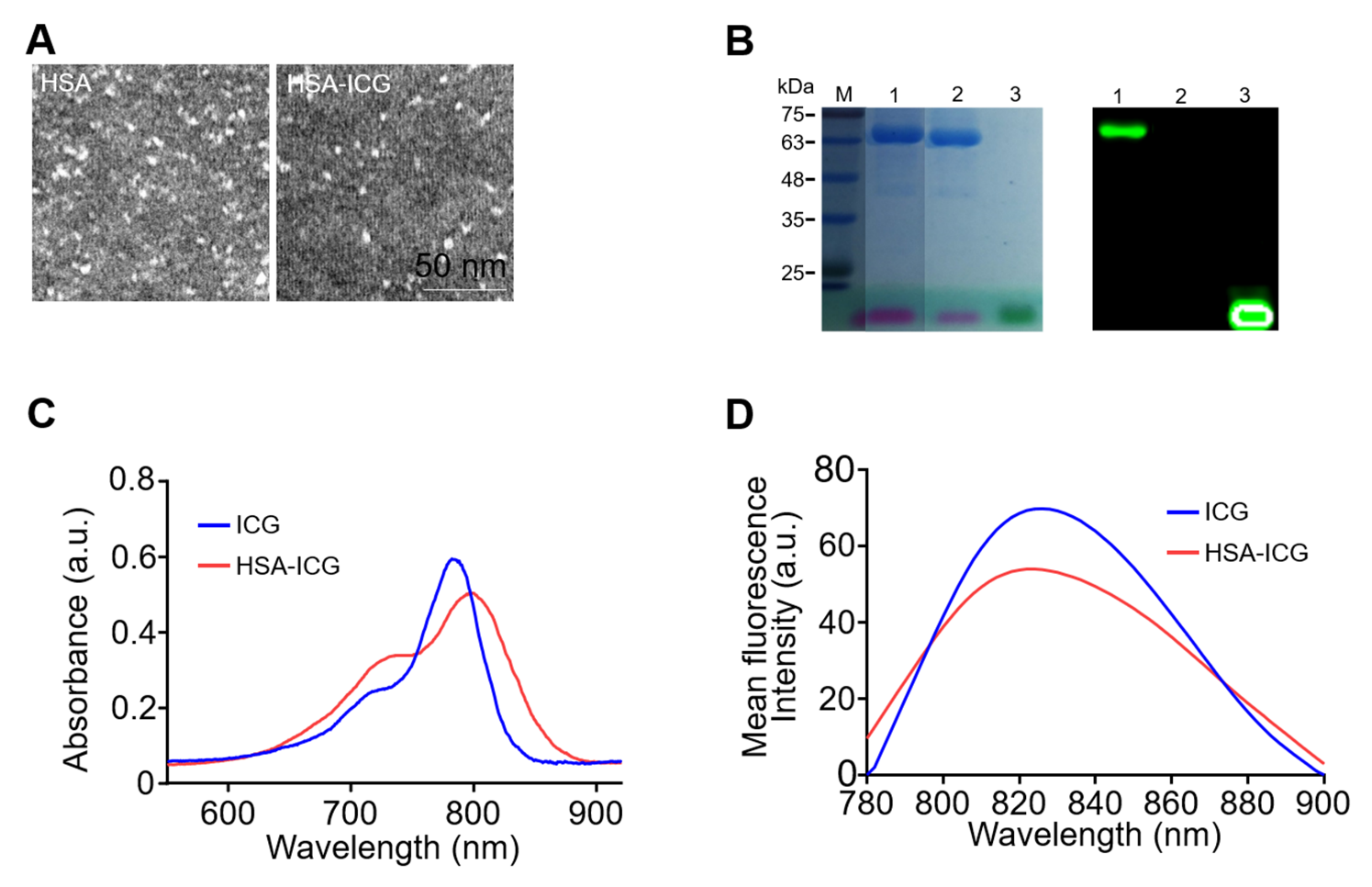


**Figure S1.** Characterization of HSA-ICG. (A) TEM image of HSA before and after conjugation with ICG. (B) SDS-PAGE analysis of HSA-ICG. The gel stained by Coomassie Brilliant Blue is shown on the left while Fluorescence image of the gel on the right. Line M, molecular weight marker, 1, HSA-ICG; 2, HSA; 3, ICG. Green color represents fluorescence signal. (C) The absorption spectra and (D) fluorescence emission spectra of free ICG and HSA-ICG.


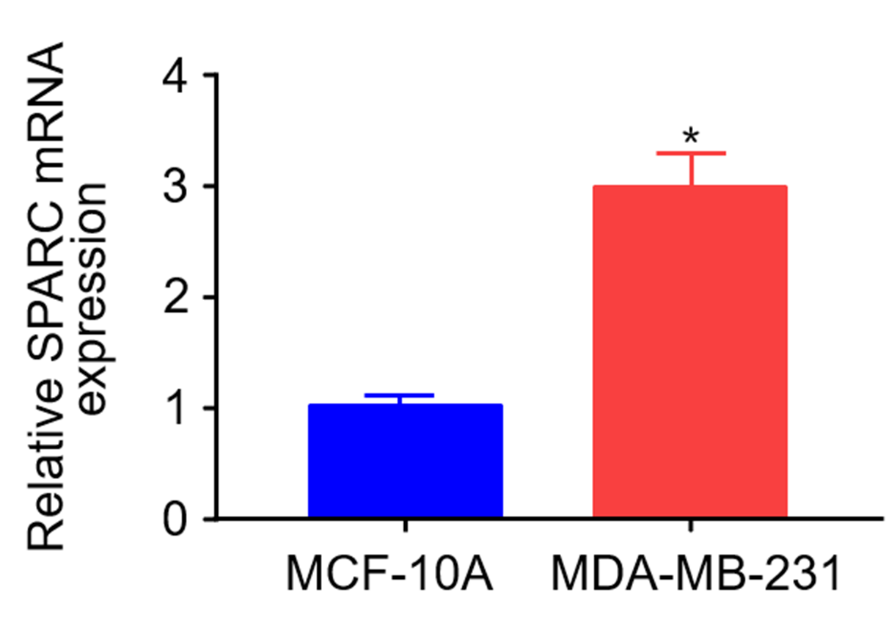


**Figure S2.** Relative mRNA expression of SPARC by reverse transcription (RT)-PCR in breast cancer MDA-MB-231cell and normal breast epithelial cell MCF 10A cell. (unpaired Student's *t* test, * *P* < 0.05)


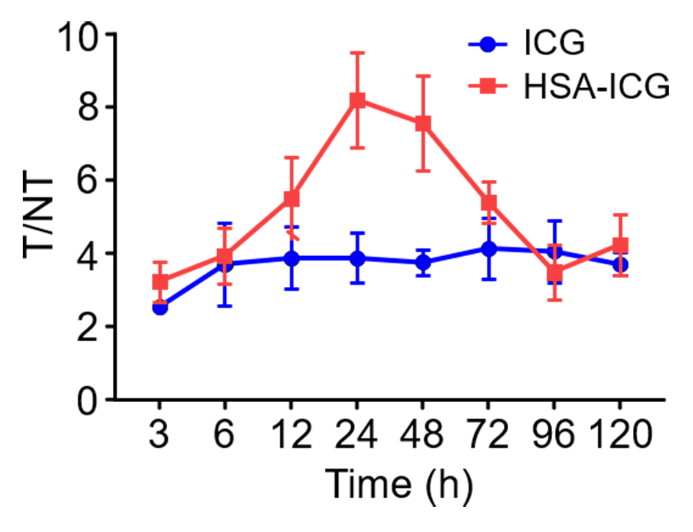


**Figure S3.** The corresponding tumor-to-background tissue ratios of HSA-ICG (top) and ICG (down). Background tissue was from the upper limb.


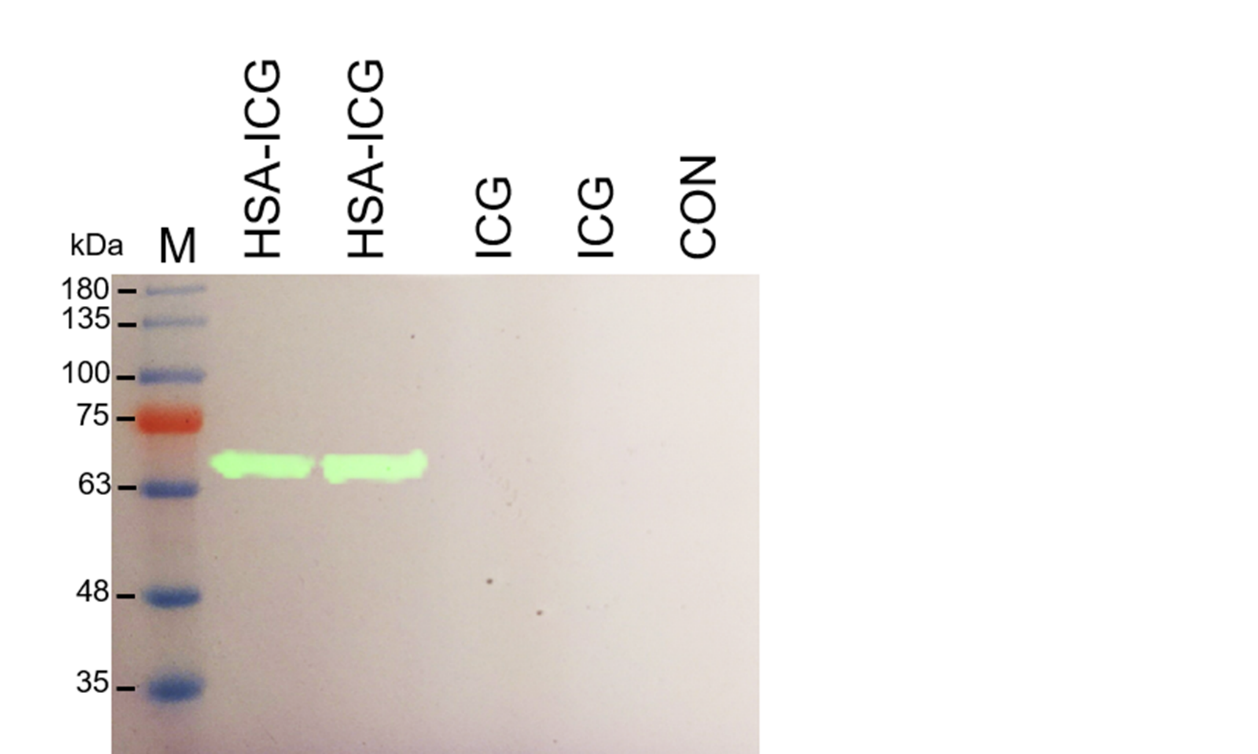


**Figure S4.** SDS-PAGE analysis of tumor lysates. Tumor lysates from HSA-ICG group, compared with ICG group and control group. The bands of tumor lysate from the HSA-ICG group were present at the same molecular weight of HSA (63-75 kDa).


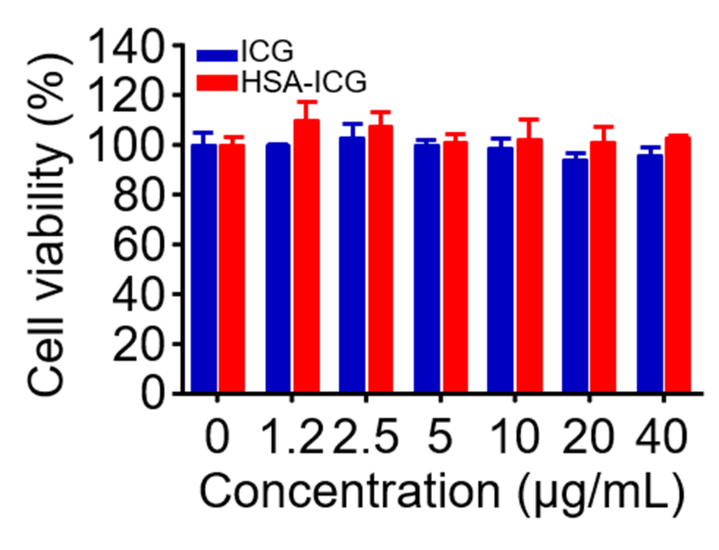


**Figure S5.** Viability of MDA-MB 231 cells incubated with HSA-ICG or ICG at various concentrations for 24 h.


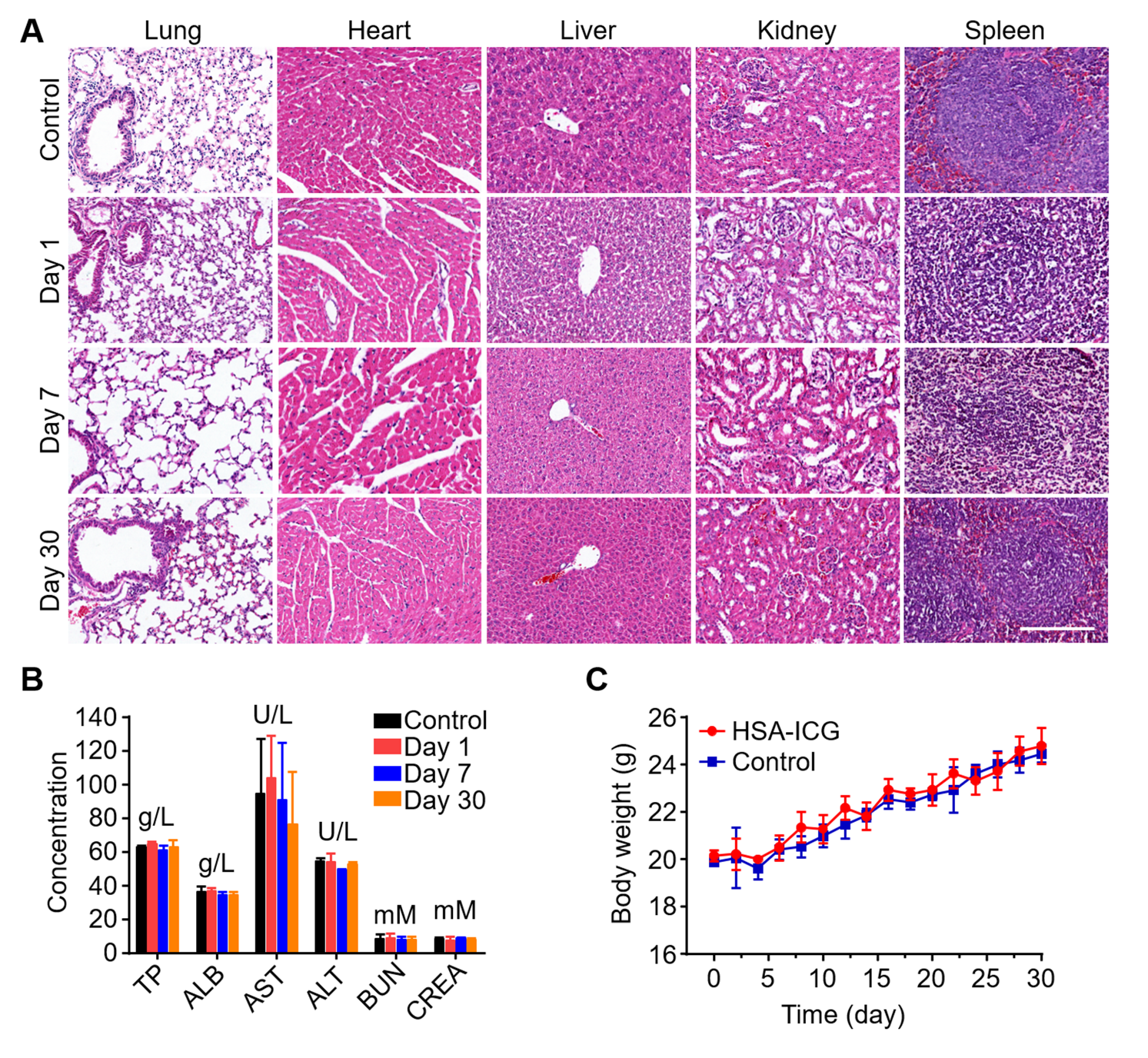


**Figure S6.** The toxicity evaluation of HSA-ICG. (a) Representative H&E stained images of the lungs, heart, liver, kidney and spleen 1, 7, 30 days after tail vein injection of HSA-ICG (containing 10 mg kg−1 ICG) or PBS (n = 3 per group). Scale bar = 200 µm. (b) Blood TP, ALB, AST, ALT, BUN and CREA 1, 7, 30 days after intravenous injection of HSA-ICG (containing 10 mg kg−1 ICG) or PBS (n = 3 per group). (c) Body weight over a span of 30 days after intravenous injection of HSA-ICG (containing 10 mg kg−1 ICG) or PBS (n = 3 per group). Mice with tail vein injection of PBS were used as the control. TP, total protein. ALB, albumin. AST, aspartate aminotransferase. ALT, alanine aminotransferase. BUN, blood urea nitrogen. CREA, creatinine.
